# Prediction of traditional Chinese medicine for diabetes based on the multi-source ensemble method

**DOI:** 10.3389/fphar.2025.1454029

**Published:** 2025-01-30

**Authors:** Bin Yang, Qingyun Chi, Xiang Li, Jinglong Wang

**Affiliations:** ^1^ School of Information Science and Engineering, Zaozhuang University, Zaozhuang, China; ^2^ Information Department, Qingdao Eighth People’s Hospital, Qingdao, China; ^3^ College of Food Science and Pharmaceutical Engineering, Zaozhuang University, Zaozhuang, China

**Keywords:** diabetes, multi-source, traditional Chinese medicine formulas, ensemble, medicinal herbs

## Abstract

**Introduction:**

Traditional Chinese medicine (TCM) prescriptions are generally formulated by experienced TCM researchers based on their expertise and data statistical methods.

**Methods:**

In order to predict TCM formulas for diabetes more accurately, this paper proposes a novel multi-source ensemble prediction method that combines machine learning ensemble techniques and multi-source data. In this method, the multi-source data contain datasets based on the components and targets (DPP-4 and GLP-1). Gradient boosting decision tree (GBDT), flexible neural tree (FNT), and Light Gradient Boosting Machine (LightGBM) algorithms are trained using these two types of datasets, respectively. The compound dataset from the TCMSP database is then used as testing data to predict and screen the active ingredients. The frequencies of occurrences of medicinal herbs corresponding to these three algorithms are obtained, each containing an active ingredient list. Finally, the frequencies of occurrences of the medicinal herbs obtained from the three algorithms using the component and target datasets are integrated to select duplicate drugs as the candidate drugs for diabetes treatment.

**Results:**

The identification results reveal that theproposed ensemble method has higher accuracy than GBDT, FNT, and LightGBM. The medicinal herbs predicted include *Lycii fructus*, *Amygdalus communis vas*, *Chrysanthemi flos*, *Hippophae fructus*, *Mori folium*, *Croci stigma*, *Maydis stigma*, *Ephedrae herba*, *Cimicifugae rhizoma*, licorice, and *Epimedii herba*, all of which have been proven effective in the treatment of diabetes.

**Discussions:**

The results of network pharmacology show that myrrha can play a role in treating diabetes through multiple targets and pathways.

## 1 Introduction

Diabetes mellitus (DM) is an endocrine metabolic disease characterized by chronic hyperglycemia, which is formed by genetic and environmental factors (Zhang Y. et al., 2024; Öztürk et al., 2017). The basic pathological characteristics of this disease are absolute or relative insufficiency of insulin secretion or decreased sensitivity of peripheral tissues to insulin, which could result in a systemic metabolic disease mainly characterized by abnormal glucose metabolism (Jr et al., 2005; Abdul-Ghani et al., 2006). Chronic hyperglycemia can cause long-term damage, dysfunction, and even failure of various organs, especially the eyes, kidneys, nerves, and cardiovascular system (Schuster et al., 2002). The clinical treatment methods include diet control (Esposito et al., 2009; Simpson et al., 1981), exercise therapy (Balducci et al., 2014), hypoglycemic drug therapy (Prasathkumar et al., 2022; Khan and Baig, 2022), and insulin therapy (Saglam and Bektas, 2023; Nkonge et al., 2023). However, in clinical practice, oral hypoglycemic drugs such as metformin, proinsulin secretagogues, and α-glucosidase inhibitors often have clinical limitations due to side effects, including gastrointestinal reactions, nutritional and metabolic disorders, allergic reactions, and liver function damage (Akinwunmi, 2020). In addition, the treatment mechanism is single. Therefore, searching for safe, effective, multi-directional, and multi-target treatment methods has become a focus of current medical research.

The overall therapeutic characteristics of single and compound Chinese medicine, with their multiple targets and effects, provide certain advantages and potential in the treatment of certain diseases (Luo et al., 2024). Traditional Chinese medicine (TCM) and its active ingredients in the prevention and treatment of diabetes are characterized by stable efficacy, fewer adverse reactions, and suitability for long-term use, which may also result in delayed complications, multi-target and multi-channel regulation of metabolic disorders, and protection of the structure and function of the pancreas, liver, kidney, and other related organs. With the acceleration of the TCM modernization process, the research on TCM prescriptions for diabetes has also attracted more attention (Chen and Dun, 2016; Zhou, 2022; Gao et al., 2024; Zhou et al., 2022; Li et al., 2022). Many TCM prescriptions, including Huanglian decoction (HLD) (Pan et al., 2020), Jiao-tai-wan (JTW) (Tang et al., 2021), Yuye Decoction (Guo et al., 2023), Roselle (Niu et al., 2022), Rhizoma coptidis (Chen et al., 2021), and Ling-Gui-Zhu-Gan (LGZG) (Long et al., 2023), have been mentioned and utilized to investigate the action mechanism in treating diabetes. These TCM prescriptions are generally formulated by experienced TCM researchers based on their expertise. However, this knowledge and experience are extensive, and the relationships between them are very complex, making it difficult to effectively analyze TCM prescriptions solely through manual labor. Therefore, data mining methods have been applied to search the TCM formulas.

Data mining can effectively induce and analyze the massive amounts of TCM data, summarize patterns, discover and solve potential problems, and provide more accurate descriptions and judgments of the relationships between various characteristic information elements of TCM, which also provide more reliable data support for TCM theory and practice (Zhang X. et al., 2024). Statistical and clustering algorithms are commonly used methods. Generally, the frequency of the presence of traditional Chinese medicinal materials in the prescriptions is counted, and a pair of medicinal materials with the highest frequency is selected for research. In order to predict TCM formulas more accurately, this paper proposes a novel prediction method of TCM against diabetes based on the multi-source ensemble method. First, the most recent literature on the treatment of the disease was collected based on disease keywords in order to construct three datasets: one based on components, another based on two targets (dipeptidyl peptidase-4 (DPP-4) and glucagon-like peptide-1 (GLP-1)), and a compound dataset from the TCMSP database. In the multi-source ensemble method, gradient boosting decision tree (GBDT), flexible neural tree (FNT), and Light Gradient Boosting Machine (LightGBM) algorithms were trained using the component and target datasets. The compound dataset from the TCMSP database is used as testing data to predict and screen the active ingredients. The frequencies of occurrences of medicinal herbs corresponding to these three algorithms are obtained, respectively, which contain the active ingredients. Finally, the frequencies of occurrences of the medicinal herbs obtained from the three algorithms using the component and target datasets are integrated to select the duplicate drugs as the candidate drugs for diabetes treatment.

## 2 Methodology

### 2.1 Data collection

#### 2.1.1 Component and target datasets

Based on keywords such as diabetes and type 2 diabetes mellitus (T2DM), the full and abbreviation names of the disease are searched in the public literature databases such as CNKI, Springer, Elsevier, NCBI, and IEEE in order to collect the literature related to the treatment and prevention of diseases with TCM prescriptions by network pharmacology. The collected literature reports are preprocessed by removing reports that use the same prescription and selecting those with a more complete analysis process. Finally, 81 literature reports have been obtained, which were published in the past 5 years. The data mining method is utilized to search for the active ingredients for treating diabetes. After deleting the duplicate ingredients, 124 ingredients were collected, which can be downloaded at https://github.com/batsicilab/Data. These ingredients have been validated by biological experiments or have a good combination with diabetes-related targets via molecular docking.

The ingredient set collected for the treatment or prevention of diabetes is designated as the positive set and is input into the DUD-E website to generate a component set unrelated to the disease (Mysinger et al., 2012). Compared to the positive samples, there are more non-related compounds generated. In order to construct the dataset with the balanced numbers of positive and negative samples, a negative sample selection algorithm is utilized to select the accurate unrelated compounds, which is described in Algorithm 1. This algorithm utilizes the Tanimoto index to evaluate the distance between two components. The Tanimoto index between compounds *C*_1_ and *C*_2_ is defined as Equation 1.

$$T\left(C_{1},C_{2}\right)=\frac{C_{1}\cap C_{2}}{C_{1}\cup C_{2}}.$$ (1)

The larger the Tanimoto index, the higher the similarity between two compounds. For each related compound, its Tanimoto index with each unrelated compound is calculated. These indexes are arranged in ascending order, and a certain proportion of unrelated samples are selected as the negative samples based on the pre-defined proportion of positive and negative samples. The specific algorithm is shown in Algorithm 1. Using Algorithm 1, the negative sample set is constructed. In addition, combined with the positive sample set, the dataset based on the component is constructed.

Algorithm 1. Negative sample selection algorithm.

**Input:** related compound set of a target gene $\left[c_{1},c_{2},\ldots ,c_{m}\right]$ ( m is the number of
   compounds) and the generated decoy set $\left[g_{1},g_{2},\ldots ,g_{n}\right]$ ( n is the number of
   decoys);
**Output:** the selected negative compound set $\left[n_{1},n_{2},\ldots ,n_{2m}\right]$

**for** *i* = 1; i≤n ; *i*++ **do**
  
$sum_{i}=0;$

  **for** *j* = 1; j≤m ; *j*++ **do**
   
$\begin{array}{l} T_{ij}=Tanimotoindex\left(g_{i},c_{j}\right);\\ sum_{i}=sum_{i}+T_{ij}; \end{array}$

  **end**
**end**
Sort the decoy set according to $\left[sum_{1},sum_{2},\ldots ,sum_{n}\right]$ ;
Select the decoys with 2m smallest Tanimoto indexes as the negative compound set.

With the same process, the target genes are collected from the literature for treating diseases, and the top 10 target genes are selected based on their occurrence frequencies. These target genes are input into BindingDB website to search for the target-related compounds (Liu et al., 2007). Target genes that do not meet the requirements were deleted based on the number of compounds collected. Finally, dipeptidyl peptidase-4 (DPP-4) and glucagon-like peptide-1 (GLP-1) are selected. These two targets have been proven to be very effective in the treatment of type 2 diabetes. GLP-1 can participate in the regulation of blood glucose homeostasis, improve islet function, and delay or even reverse the progression of type 2 diabetes through multiple pathways. DPP-4 inhibitors can effectively improve blood glucose levels and promote insulin secretion in the body. The compounds related to DPP-4 and GLP-1 are considered the positive sample sets, with their numbers being 248 and 89, respectively. The negative sample set for each target gene is obtained using Algorithm 1. In addition, the datasets based on DPP-4 and GLP-1 are obtained.

#### 2.1.2 Compound dataset from the TCMSP database

All compounds are collected from the TCMSP database, totaling 13,144 (Ru et al., 2014). Oral bioavailability (OB) represents the speed and degree of the absorption of TCM in human circulation. Drug-like (DL) properties represent the similarity between a compound and a known drug. Thus, OB ≥ 25% and DL ≥ 0.15 are set, which are utilized to screen active ingredients. According to OB and DL screening, a total of 2,376 compounds are obtained from the TCMSP database, which are utilized as a compound set for future analysis.

### 2.2 Classification methods

#### 2.2.1 Gradient boosting decision tree

GBDT is a supervised learning algorithm that combines gradient enhancement and decision tree (Zhuang et al., 2017). The main idea is to gradually reduce the residuals through a continuous iterative process and construct multiple regression decision trees through gradient optimization. Finally, the conclusions of all regression trees are summarized to form the final model, with the aim of simultaneously reducing the variance and bias of the model. In addition to the strong interpretability of tree models and the effective processing ability of mixed models, its other advantages are high prediction accuracy, strong robustness, and the ability to flexibly handle various types of data.

In GBDT, each iteration aims to minimize the current loss function. After each iteration, the updated model is defined as Equation 2.

$$M_{k}\left(x\right)=M_{k-1}\left(x\right)+\arg\min_{h}\sum_{i=1}^{n}L\left[y_{i},M_{k-1}\left(x_{i}\right)+h_{i}\left(x\right)\right],$$ (2)

where $M_{k}\left(x\right)$ is the prediction result of the decision tree generated for the *k*th iteration, n is the number of samples, $L\left[y_{i},M_{k-1}\left(x_{i}\right)+h_{i}\left(x\right)\right]$ is the loss function, $y_{i}$ is the actual value of the *i*th sample, and $h_{i}\left(x\right)$ is a weak learning function.

Through continuous iterations, the final residual of the model approaches 0, which satisfies the convergence condition. Finally, the decision tree generated by the iteration is linearly combined through an additive model to obtain the prediction result. The final prediction result of GBDT can be calculated as Equation 3.

$$M\left(x\right)=\sum_{k=1}^{M}M_{k}\left(x\right),$$ (3)

where M is the number of decision trees.

#### 2.2.2 Flexible neural tree model

**FIGURE 1:**
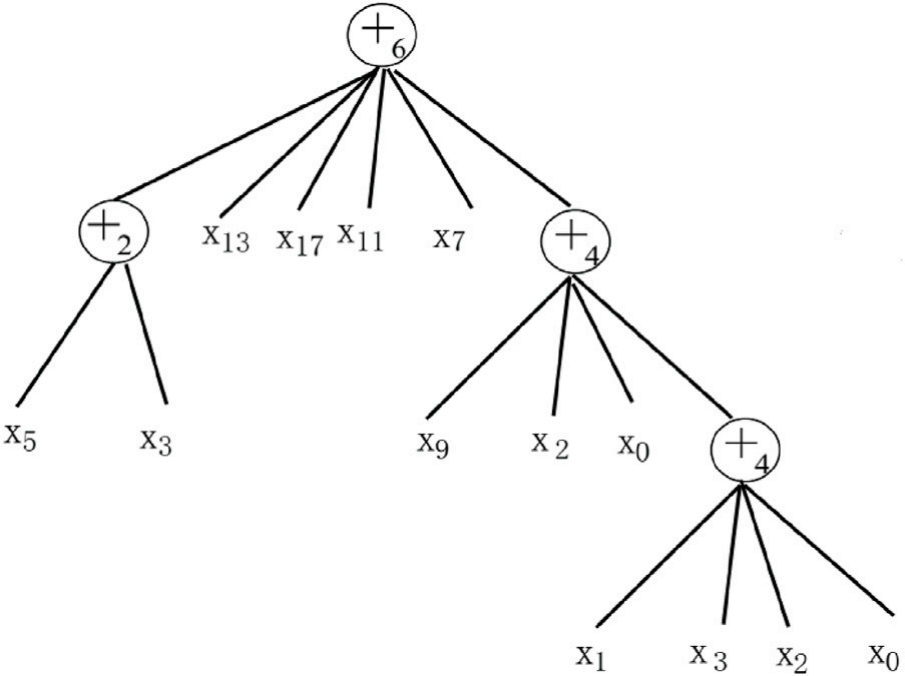
Example of the flexible neural tree model.

FNT can automatically determine the network structure and allow cross-layer connections between different nodes, making it a high-performance deep neural network structure (Chen et al., 2006). An example of FNT is depicted in Figure 1. During the construction of FNT, two main parts need to be determined, namely, the structure of the tree and the leaf nodes of the tree. So, FNT has two basic instruction sets, namely, the neuron instruction set (*F*) and the terminal instruction set (*T*). The neural instruction set determines the tree structure of FNT, which takes the basic form as +_*i*_. The terminal instruction set determines the input vector of FNT. The sets of neuron and terminal instructions can be represented as Equation 4.

$$\left\{\begin{array}{l} F=\left\{{+}_{2},{+}_{3},\ldots ,{+}_{n}\right\}\\ T=\left\{x_{1},x_{2}\ldots ,x_{m}\right\} \end{array}\right.,$$ (4)

where *n* represents the number of child nodes owned by the non-leaf node +_*n*_. In other words, the node will have *n* input features. The output of the non-leaf node +_*n*_ is calculated as Equation 5.

$$\left\{\begin{array}{l} o_{n}=\sum_{j=1}^{n}w_{j}x_{j},\\ y_{n}=f\left(a,b,o_{n}\right)=e^{-{\left(\frac{o_{n}-a}{b}\right)}^{2}}, \end{array}\right.$$ (5)

where $f\left(\cdot \right)$ denotes the activation function, a and b are the parameters of the function, $x_{j}$ is the input variable, and $w_{j}$ is the corresponding weight of the input variable.

During the evolutionary process, first, an FNT tree and a set of corresponding parameters are randomly generated and then the structure of the tree is optimized. After finding the optimal tree structure, the particle swarm optimization (PSO) algorithm is used to optimize the parameters of the connection weights in the tree structure. In addition, a complete and optimal flexible neural tree is ultimately generated.

#### 2.2.3 LightGBM

LightGBM utilizes a decision tree based on the histogram, which could discretize continuous eigenvalue data into histograms (Gao and Balyan, 2022). The algorithm adopts Gradient-based One-Side Sampling (GOSS) technology, which is utilized to sample the data with small gradients based on sample gradients, while the samples with large gradients are retained for sampling and splitting selection in order to reduce the number of samples required for calculation. GOSS could improve the training speed and memory efficiency of the model. Exclusive Feature Bundling (EFB) technology is utilized to bundle mutually exclusive features into a single feature in order to reduce the number of features in training samples, thereby improving training efficiency and accuracy. It is suitable for training high-dimensional and sparse data.

### 2.3 Prediction of TCM for diabetes based on the multi-source ensemble method

**FIGURE 2:**
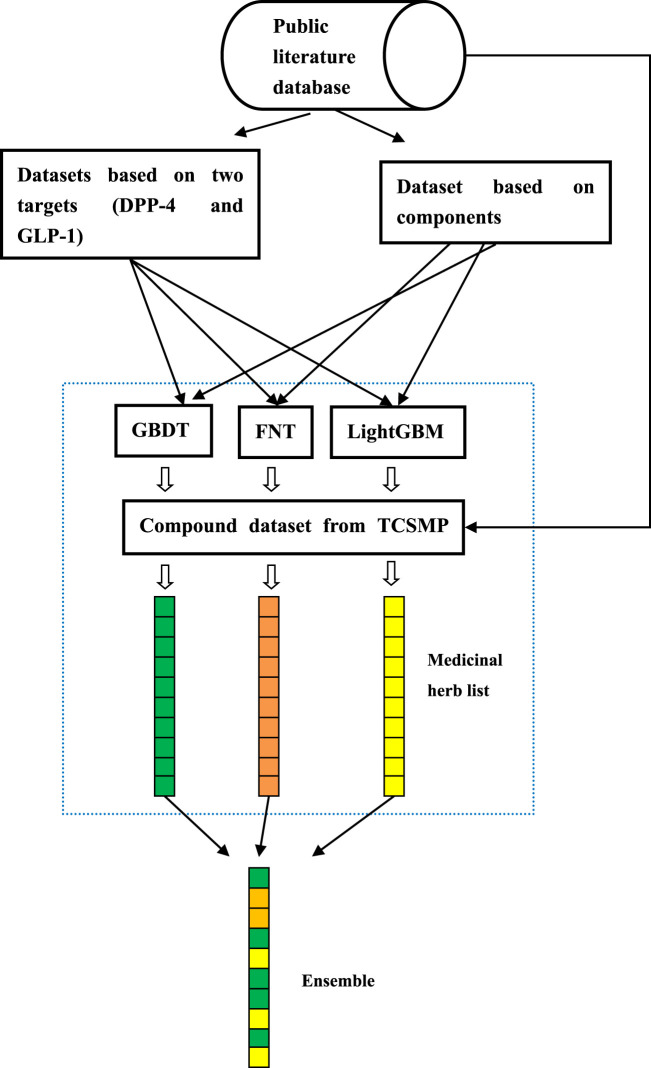
Prediction flowchart of TCM for diabetes based on the multi-source ensemble method.

In this part, a novel prediction method of TCM in treating diabetes based on a multi-source ensemble method is proposed; the flowchart of this method is depicted in Figure 2.

(1) Dataset construction. The latest literature on the treatment of diabetes is collected using disease-related keywords in order to construct a dataset based on components, another based on targets (DPP-4 and GLP-1), and a compound dataset from the TCMSP database. Molecular descriptors are utilized to extract the feature vectors for each compound in these datasets.
(2) Prediction based on the multi-source ensemble method. The datasets based on targets and components are input into three algorithms (GBDT, FNT, and LightGBM) for training to obtain the optimal models. Then, as the testing data, the compound dataset from the TCMSP database is predicted, and all the components are scored. The compounds above 0.5 are considered active ingredients. The annotation information in the TCMSP database is utilized to obtain the medicinal herbs that contain the active ingredients. The frequencies of occurrences of medicinal herbs corresponding to these three algorithms are obtained, respectively. Finally, for each classification algorithm, the top 20 ranked medicinal herbs are selected as the medicinal herb list of each algorithm. The medicinal herb lists obtained from three methods are integrated. In addition, the herbs with a frequency of occurrence equal to or greater than 2 are selected as the candidate drugs for diabetes treatment.

## 3 Results

**FIGURE 3:**
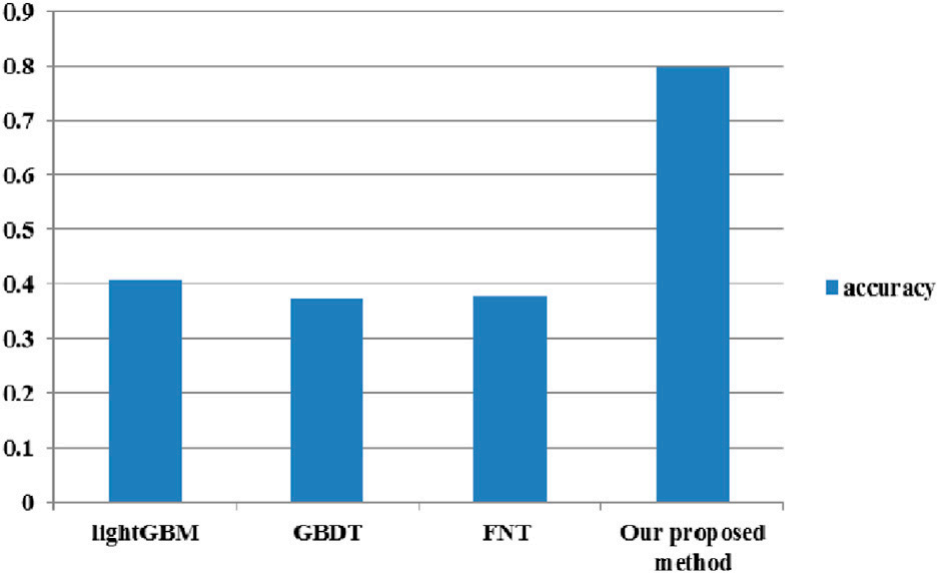
Accuracy performance of the proposed method and three classification methods.

In this section, the dataset based on components includes 124 positive and 248 negative samples, while the dataset based on the two targets includes 337 positive and 1,011 negative samples. In GBDT and lightGBM, the tree-structured Parzen estimator (TPE) is utilized to optimize the hyperparameters of these methods. In FNT, the model and hybrid optimization algorithm contain more hyperparameters. In addition, the optimization algorithm could be utilized to iteratively search the optimal FNT model. Therefore, the hyperparameters of FNT are selected by experience. In our method, the numbers of the compounds predicted by GBDT, lightGBM, and FNT are 655, 183, and 872, respectively. The numbers of the predicted herbs are 399, 226, and 415, respectively. Single hypoglycemic herbs are collected from the 2020 edition of “Chinese Pharmacopoeia” (Part 1) and Chinese Materia Medica (third edition) as a standard set (Jing et al., 2024). The accuracy performance of the proposed method and three classification methods is depicted in Figure 3. Figure 3 shows that our proposed method could obtain the highest accuracy performance, which is 96.5% higher than that of lightGBM, 112.8% higher than that of GBDT, and 110% higher than that of FNT.

The herbs with a frequency of occurrence equal to or greater than 2 include *Lycii fructus*, *Amygdalus communis vas*, *Chrysanthemi flos*, *Hippophae fructus*, *Mori folium*, *Croci stigma*, *Maydis stigma*, myrrha, *Microctis folium*, *Forsythiae fructus*, *Ephedrae herba*, *Cimicifugae rhizoma*, licorice, and *Epimedii herba*.

## 4 Discussions

According to the 2020 edition of the “Chinese Pharmacopoeia” (Part 1) and Chinese Materia Medica (third edition), *Lycii fructus*, *Amygdalus communis vas*, *Chrysanthemi flos*, *Hippophae fructus*, *Mori folium*, *Croci stigma*, *Maydis stigma*, *Ephedrae herba*, *Cimicifugae rhizoma*, licorice, and *Epimedii herba* have been proven to be single hypoglycemic herbs. *Forsythiae fructus* exhibits anti-tumor, antiviral, and hypoglycemic effects (Liu et al., 2020). Xu et al. found that Forsythia glycoside, *Forsythia suspensa* glycoside A, and Rosin-β-D-fructose extracted from *Forsythiae fructus* leaves can increase glucose uptake in 3T3-L1 adipocytes under insulin resistance by activating the PI3K/Akt signaling pathway, thereby increasing their insulin sensitivity (Xu et al., 2019). Some TCM prescriptions for treating T2DM include *Microctis folium*, such as Shuzheng granules. The extract of myrrha has a direct stimulating effect on insulin secretion in β-cell lines and primary pancreatic islets under low- and high-stimulation concentrations, indicating that myrrha can regulate insulin release in β-cells without being affected by glucose levels (Al-Romaiyan et al., 2021). Preliminary research and analysis indicate that the predicted drugs had good effects on the treatment of diabetes and its complications, thereby validating the effectiveness of our proposed prediction method.

In order to further verify the effectiveness of our ensemble method, the network pharmacology method will be utilized to explore the mechanism of myrrha in the treatment of diabetes.

### 4.1 Candidate target of myrrha in the treatment of diabetes

Through the Traditional Chinese Medicine Systems Pharmacology Database and Analysis Platform (TCMSP), myrrha is utilized as the keyword for search, and according to oral bioavailability (OB) > 30 and drug-like (DL) properties > 0.18, 22 effective ingredients of myrrha are obtained. These effective ingredients are input into the SwissTargetPrediction Database (http://www.swis-stargetprediction.ch/) in order to obtain drug targets by removing duplicate target genes. With diabetes mellitus as the keyword, the diabetes-related targets are searched in the GeneCards database. By intersecting drug targets with disease targets, a total of 442 candidate targets are obtained.

### 4.2 Construction of the protein–protein interaction network of candidate targets

**FIGURE 4:**
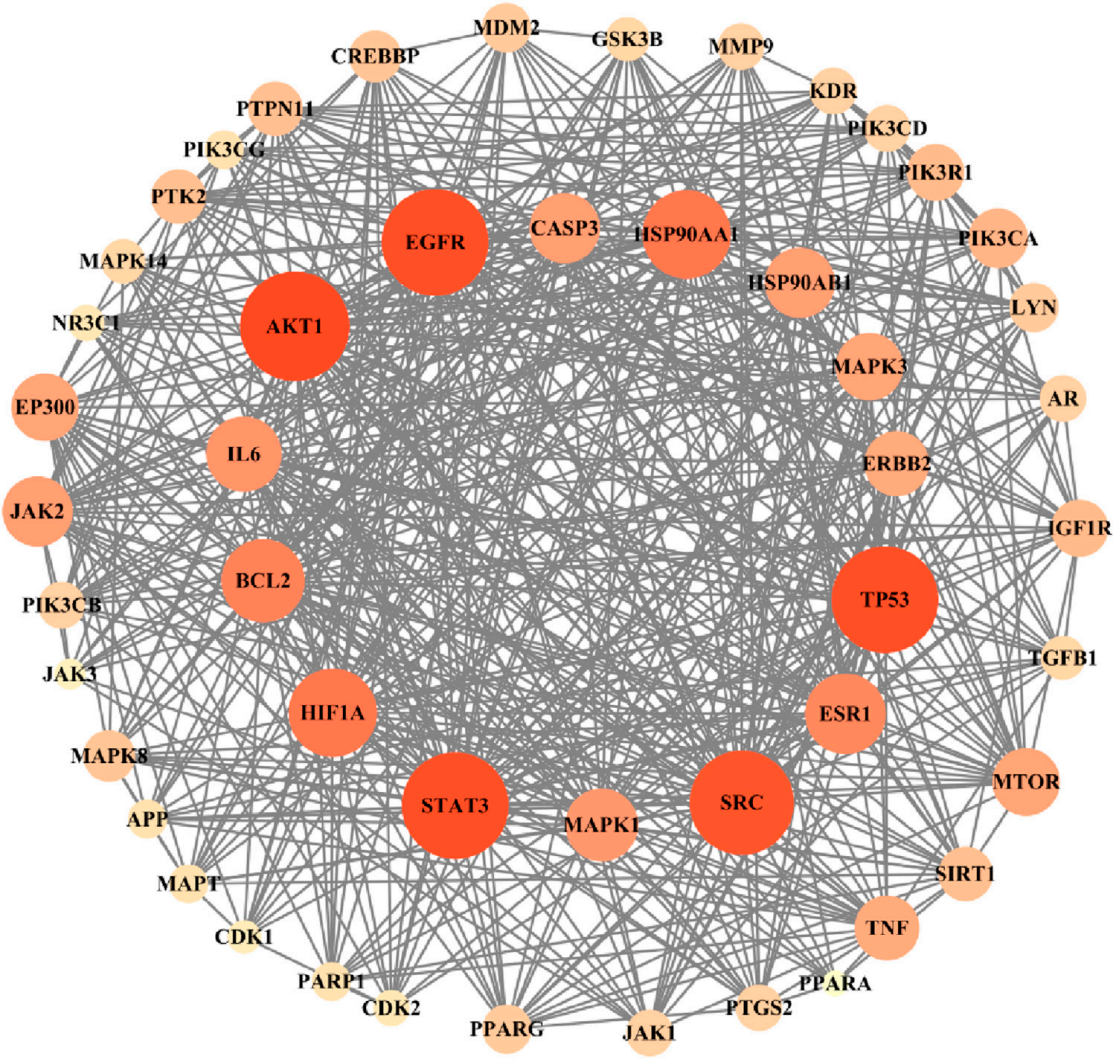
Protein–protein interaction network of potential targets of myrrha for diabetes treatment.

A total of 442 candidate action targets are imported into the STRING database, and the protein–protein interaction (PPI) network of potential targets of myrrha for diabetes treatment is obtained, which includes 442 nodes (target proteins) and 2,421 edges (protein interactions). According to the degree ranking, TP53, STAT3, SRC, HSP90AA1, EGFR, AKT1, IL6, PIK3CA, BCL2, and HIF1A have high degree values, which may be key targets for the treatment of diabetes. In addition, TNF, PIK3R1, ESR1, ERBB2, JAK2, and MAPK1 may be effective targets. The PPI network containing 50 targets with the largest degrees is depicted in Figure 4. The larger the node and the darker the orange color, the higher the corresponding degree value, indicating that there are more targets in the predicted disease-related targets that can effectively interact with this target.

### 4.3 KEGG enrichment analysis

**FIGURE 5:**
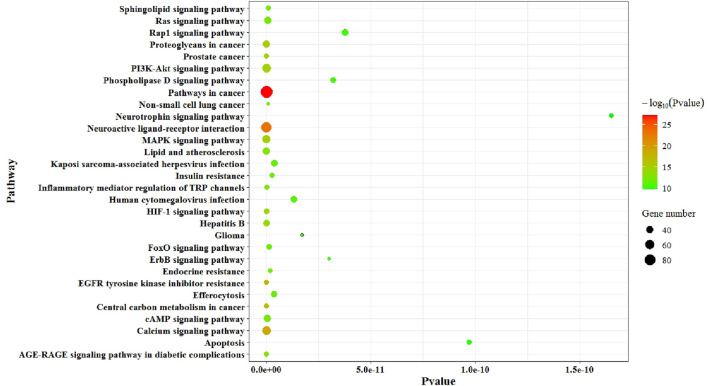
Bubble plot of the KEGG pathway enrichment analysis.

Potential targets are imported into the DAVID database (https://david.ncifcrf.gov/) in order to perform KEGG pathway enrichment analysis. The bubble plot is drawn with the top 30 pathways according to the *p*-value, as shown in Figure 5. Figure 5 shows that KEGG analysis mainly involves the following main pathways: pathways in cancer, neuroactive ligand–receptor interactions, the calcium signaling pathway, EGFR tyrosine kinase inhibitor resistance, central carbon metabolism in cancer, proteoglycans in cancer, the MAPK signaling pathway, the PI3K–Akt signaling pathway, prostate cancer, the HIF-1 signaling pathway, hepatitis B, the AGE–RAGE signaling pathway in diabetic complications, lipid and atherosclerosis, inflammatory mediator regulation of TRP channels, the cAMP signaling pathway, the Ras signaling pathway, non-small cell lung cancer, the sphingolipid signaling pathway, the FoxO signaling pathway, endocrine resistance, insulin resistance, efferocytosis, Kaposi’s sarcoma-associated herpesvirus infection, human cytomegalovirus infection, glioma, the ErbB signaling pathway, the phospholipase D signaling pathway, the Rap1 signaling pathway, apoptosis, and the neurotrophin signaling pathway.

### 4.4 Construction of the “component–target–pathway” network

**FIGURE 6:**
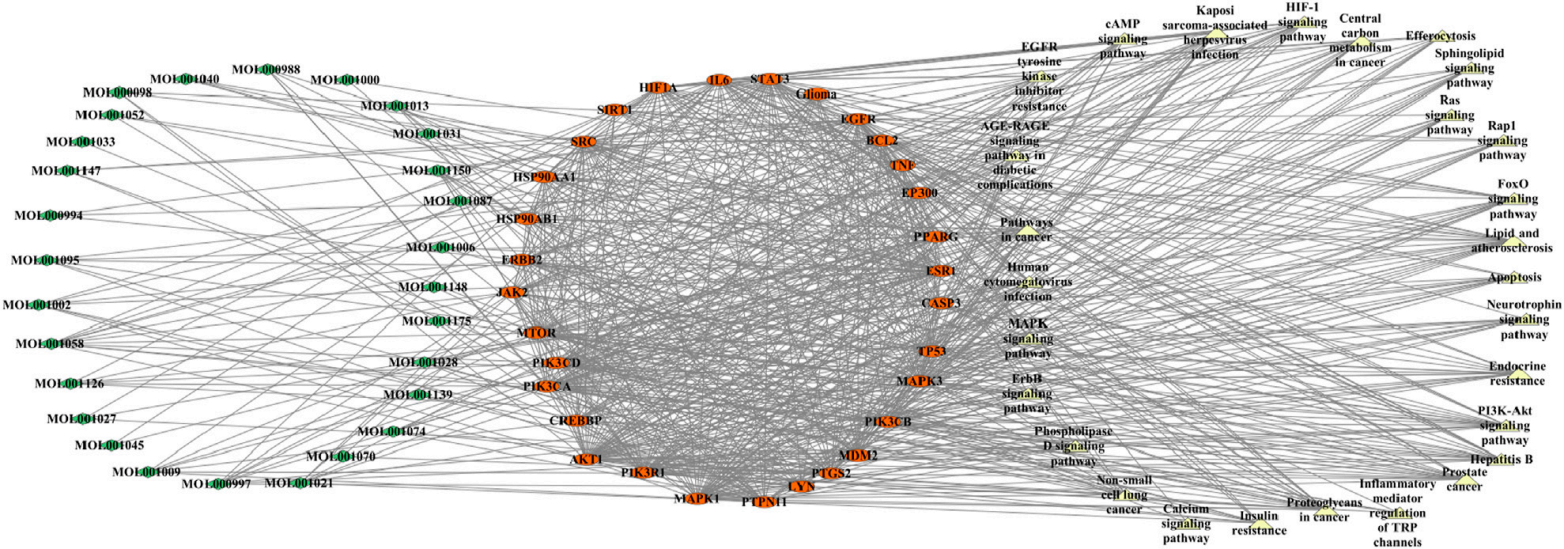
“Component–target–pathway” network.

Due to a large number of predicted potential targets, the targets with the top 30° values in the PPI network are selected to participate in the construction of the “component–target–pathway” network, as shown in Figure 6. The network contains 87 nodes (28 blood components, 30 targets, and 29 pathways) and 767 edges. The higher the degree value, the more the other nodes connected to a node, indicating that the node has a higher contribution rate in the network. By analyzing the network, the components such as MOL001058 (picropolygamain), MOL000997 (guggulsterol V), MOL001150 (3β-acetoxy-16β-hydroxydammar-24-ene), MOL001002 (ellagic acid), MOL001009 (guggulsterol-VI), MOL001021 (7β,15β-dihydroxypregn-4-ene-3,16-dione), and MOL001013 (mansumbinoic acid) have the higher degree values and are all connected to multiple targets. The targets with higher degree values include AKT1, MAPK3, EGFR, MAPK1, PIK3CA, TP53, SRC, PIK3R1, and PIK3CB. Pathways with higher degree values not only involve cancer pathways but also involve lipids and atherosclerosis, Kaposi’s sarcoma-associated herpesvirus infection, the PI3K–Akt signaling pathway, hepatitis B, the HIF-1 signaling pathway, endocrine resistance, EGFR tyrosine kinase inhibitor resistance, the FoxO signaling pathway, the AGE–RAGE signaling pathway in diabetic complications, apoptosis, the ErbB signaling pathway, the sphingolipid signaling pathway, the neurotrophin signaling pathway, and insulin resistance.

### 4.5 Molecular docking

**FIGURE 7:**
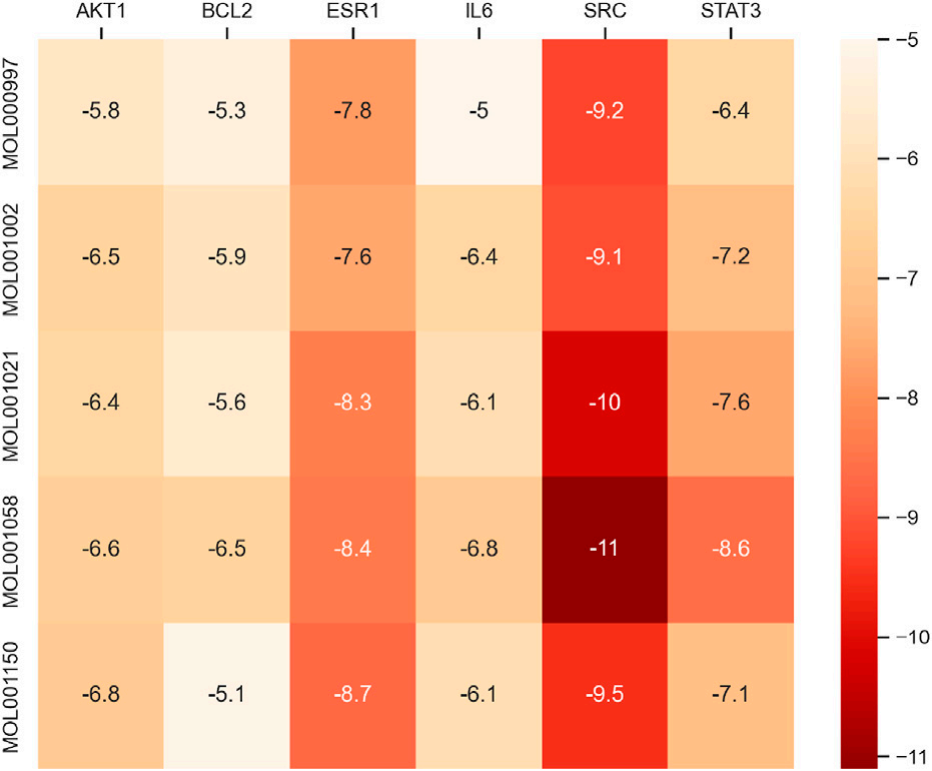
Binding energy heatmap of molecular docking.

**FIGURE 8:**
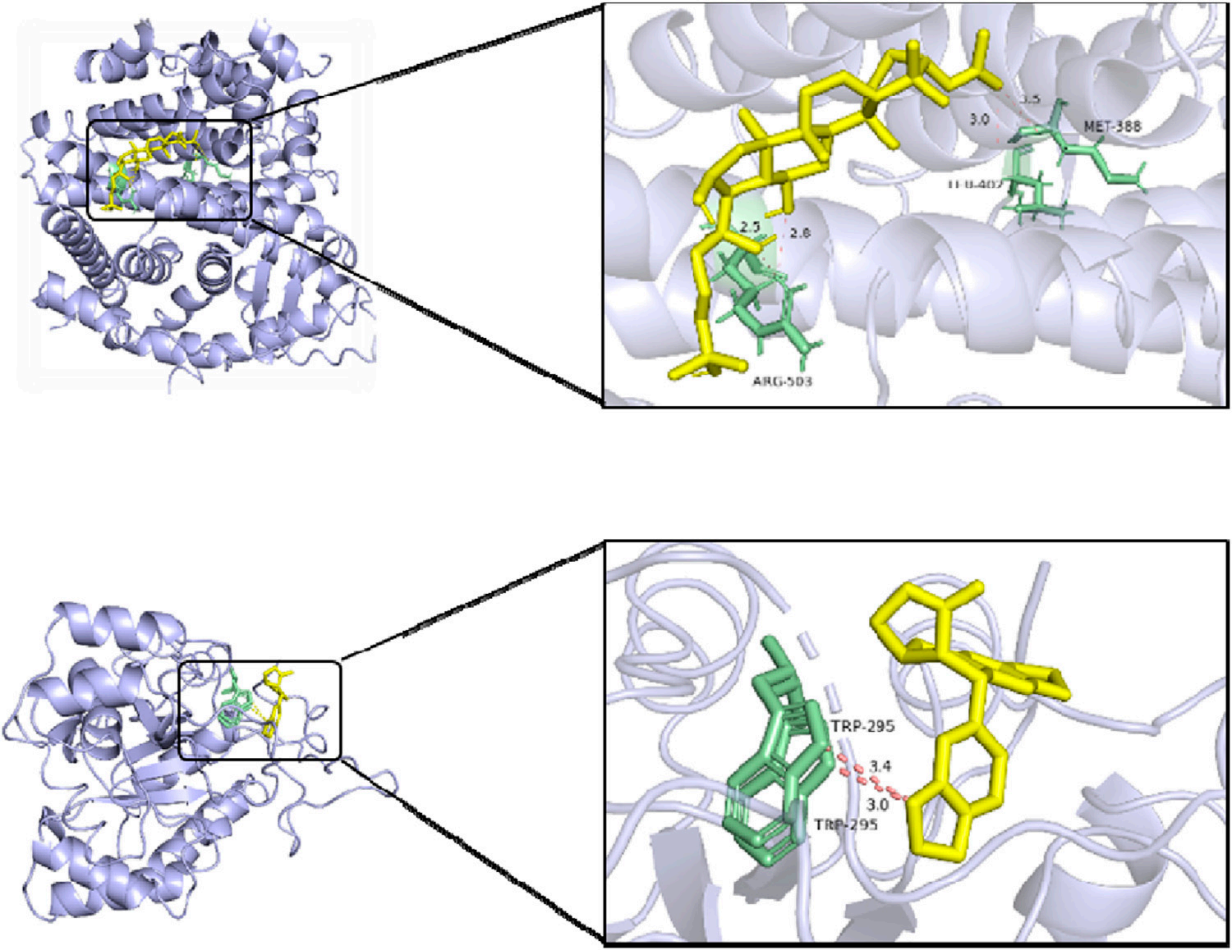
Docking models of SRC with MOL001058 (top) and ESR1 with MOL001150 (bottom).

According to the abovementioned network pharmacology analysis, the target protein with the highest median value in the PPI network was identified, and five primary active components of myrrha in the treatment of diabetes were screened out. They are MOL001058 (picropolygamain), MOL000997 (guggulsterol-V), MOL001150 (3β-acetoxy-16β-hydroxydammar-24-ene), MOL001002 (ellagic acid), and MOL001021 (7β,15β-dihydroxypregn-4-ene-3,16-dione). The MOL format files of six molecules (SRC, BCL2, STAT3, ESR1, AKT1, and IL6) were acquired from the TCMSP database and used as candidate small-molecule ligands in molecular docking. 1US0, 6HOI, 6NJS, 7NFB, 8R5K, and 5ZO6 were obtained from the PDB database as receptor protein structures for molecular docking. The binding energy heatmap of molecular docking is shown in Figure 7. The docking models of SRC with MOL001058 and ESR1 with MOL001150 are shown in Figure 8. The results indicate that most of the active components of myrrha have strong binding activity to diabetes-related targets and have high potential biological activity.

### 4.6 Result analysis

In this paper, we predicted the mechanism of myrrha in treating diabetes through network pharmacology and predicted that TP53, STAT3, SRC, AKT1, IL6, TNF, and ESR1 might be the potential gene targets of myrrha in treating diabetes.

TP53 is a tumor suppressor protein that plays an important regulatory role in cancer progression while directly regulating glycolysis and gluconeogenesis to affect glucose levels (Minamino et al., 2009). After DNA damage, TP53 can reprogram the energy generation strategy of cells from glycolysis to mitochondrial respiration (or oxidative phosphorylation) to inhibit the development of diabetes (Kruiswijk et al., 2015). STAT3 may be involved in nutrition induction and cytokine-induced insulin resistance (Mashili et al., 2013). SRC could regulate insulin secretion and glucose metabolism by affecting the subcellular localization of glucokinase in pancreatic β cells (Sato et al., 2016). AKT1 is a serine/threonine protein kinase that is an important downstream target of the insulin signaling pathway. It can regulate the body’s acquisition of glucose, maintain blood glucose stability, regulate glucose transport pathways, and play an important role in regulating pancreatic islet quality (Millischer et al., 2020). IL-6 has a hormone-like effect. It enters the bloodstream after being released from tissue, regulates the glucose metabolism and lipid metabolism of the body, provides a self-protection immune response caused by insulin resistance, and improves the formation of diabetes from insulin resistance. Tumor necrosis factor (TNF) is often secreted by macrophages and promotes insulin resistance and reduces insulin sensitivity by interfering with the insulin signaling pathway. TNF inhibition has been suggested for the treatment and prevention of T2DM (Ji et al., 2019; Trinh et al., 2019). ESR1 is a regulator of blood glucose homeostasis, which can affect blood glucose homeostasis by regulating the estrogen level and plays an important role in pancreatic β-cell function and survival, participating in the pathological and physiological processes of obesity, insulin resistance, and diabetes (Gregorio et al., 2021; Xie et al., 2024; Liu et al., 2023).

KEGG enrichment pathway analysis shows that neuroactive ligand–receptor interaction, the calcium signaling pathway, EGFR tyrosine kinase inhibitor resistance, the PI3K–Akt signaling pathway, prostate cancer, the HIF-1 signaling pathway, hepatitis B, the AGE–RAGE signaling pathway in diabetic complications, lipid and atherosclerosis, the cAMP signaling pathway, the Ras signaling pathway, the sphingolipid signaling pathway, the FoxO signaling pathway, endocrine resistance, and insulin resistance are important pathways related to the disease regulation of diabetes. Research shows that there is a significant difference in the expression of genes related to the neuroactive ligand–receptor interaction signal pathway between T2DM patients and normal subjects (Das and Rao, 2007). The cAMP signaling pathway (PKA system) is a type of cyclic nucleotide system that regulates biological cell activity when cells are stimulated (Tian et al., 2022). Ginseng polysaccharide can improve the progression of renal fibrosis in diabetic mice by inhibiting the activation of cAMP/PKA/CREB signaling pathways (Huang et al., 2018). The dysfunction of the PI3K/AKT signaling pathway and downstream target proteins can cause abnormal glucose and lipid metabolism, leading to insulin resistance and playing an important role in the occurrence and development of insulin resistance (Wei et al., 2022). The HIF-1 signaling pathway is a key signaling pathway that affects metabolic diseases. The imbalance of signal transmission of this pathway will lead to the obstacle of the adaptive response of pancreatic islets, the retina, and other tissues to hypoxia, further promoting the occurrence and development of diabetes and its complications (Wei et al., 2022; Catrina and Zheng, 2021). The KEGG enrichment pathway also refers to insulin resistance (IR). IR is a phenomenon in which the biological effects of insulin are lower than normal levels, resulting in delayed blood glucose reaching tissue cells for processing. Due to the existence of IR, blood glucose cannot be effectively used. Therefore, IR is the main pathogenesis of T2DM.

To sum up, myrrha can play a role in treating diabetes through multiple targets and pathways. Using network pharmacology, this study constructed a “component–target–pathway” network of myrrha for diabetes treatment and predicted its targets and pathways, which could provide a theoretical basis for the in-depth study of the molecular mechanism of myrrha against diabetes.

## 5 Conclusion

In this paper, a prediction method of TCM for diabetes is proposed based on the multi-source ensemble method. The medicinal herbs identified by our method include *Lycii fructus*, *Amygdalus communis vas*, *Chrysanthemi flos*, *Hippophae fructus*, *Mori folium*, *Croci stigma*, *Maydis stigma*, Myrrha, *Microctis folium*, *Forsythiae fructus*, *Ephedrae herba*, *Cimicifugae rhizoma*, licorice, and *Epimedii herba. Lycii fructus*, *Amygdalus communis vas*, *Chrysanthemi flos*, *Hippophae fructus*, *Mori folium*, *Croci stigma*, *Maydis stigma*, *Ephedrae herba*, *Cimicifugae rhizoma*, licorice, and *Epimedii herba* have been proven to be effective in the treatment of diabetes. The paper also utilizes network pharmacology to explore the mechanism of myrrha in the treatment of diabetes. In addition, the results show that myrrha can play a role in treating diabetes through multiple targets and pathways. Thus, the paper proposes an effective prediction method of TCM in the treatment of diabetes. In the future, our method will be utilized to predict TCM formulations for other diseases.

## Data Availability

The raw data supporting the conclusions of this article will be made available by the authors, without undue reservation.
